# Roles of MRI evaluation of pelvic recurrence in patients with rectal cancer

**DOI:** 10.1186/s13244-024-01842-1

**Published:** 2024-11-11

**Authors:** Patricia Perola Dantas, Verônica Botelho Teixeira, Carlos Frederico Sparapan Marques, Gerda Feitosa Nogueira, Cinthia D. Ortega

**Affiliations:** 1 Instituto do Cancer do Estado de São Paulo do Hospital das Clinicas ICESP/HCFMUSP, Department of Radiology, Faculdade de Medicina, Universidade de Sao Paulo, São Paulo, SP Brasil; 2 Colorectal Division, Instituto do Cancer do Estado de São Paulo do Hospital das Clinicas ICESP/HCFMUSP, Department of Gastroenterology and Nutrology, Faculdade de Medicina, Universidade de Sao Paulo, São Paulo, SP Brasil; 3 Instituto de Radiologia, Hospital das Clinicas HCFMUSP, Department of Radiology, Faculdade de Medicina, Universidade de Sao Paulo, São Paulo, SP Brasil

**Keywords:** Rectal cancer, Pelvic recurrence, Magnetic resonance imaging

## Abstract

**Abstract:**

Developments in the multidisciplinary treatment of rectal cancer with advances in preoperative magnetic resonance imaging (MRI), surgical techniques, neoadjuvant chemoradiotherapy, and adjuvant chemotherapy have had a significant impact on patient outcomes, increasing the rates of curative surgeries and reducing pelvic recurrence. Patients with pelvic recurrence have worse prognoses, with an impact on morbidity and mortality. Although local recurrence is more frequent within 2 years of surgical resection of the primary tumor, late recurrence may occur. Clinical manifestations can vary from asymptomatic, nonspecific symptoms, to pelvic pain, bleeding, and fistulas. Synchronous metastatic disease occurs in approximately 50% of patients diagnosed with local recurrence. MRI plays a crucial role in posttreatment follow-up, whether by identifying viable neoplastic tissues or acting as a tool for therapeutic planning and assessing the resectability of these lesions. Locally recurrent tissues usually have a higher signal intensity than muscle on T2-weighted imaging. Thus, attention is required for focal heterogeneous lesions, marked contrast enhancement, early invasive behavior, and asymmetric appearance, which are suspicious for local recurrence. However, postsurgical inflammatory changes related to radiotherapy and fibrosis make it difficult to detect initial lesions. This study therefore aimed to review the main imaging patterns of pelvic recurrence and their implications for the surgical decision-making process.

**Critical relevance statement:**

MRI plays a crucial role in the posttreatment follow-up of rectal cancer, whether by identifying viable neoplastic tissues or by acting as a tool for therapeutic planning. This study reviewed the main imaging patterns of pelvic recurrence.

**Key Points:**

- MRI aids in surgical planning and the detection of pelvic recurrence and postoperative complications.
- Being familiar with surgical techniques enables radiologists to identify expected MRI findings.
- Patterns of rectal cancer recurrence have been categorized by pelvic compartments.
- Neoplastic tissue may mimic postsurgical and postradiotherapy changes.
- Resectability of pelvic recurrence is highly related to lesion location.

**Graphical Abstract:**

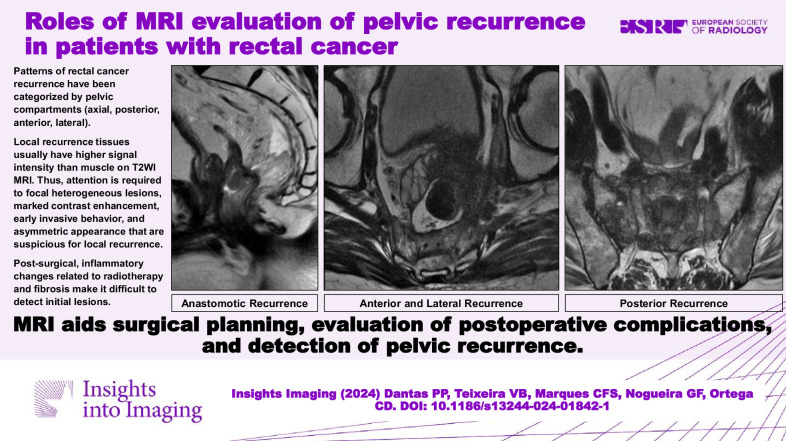

## Introduction

Developments in the multidisciplinary treatment of rectal cancer with advances in preoperative magnetic resonance imaging (MRI), surgical techniques, neoadjuvant chemoradiotherapy (CRT), and adjuvant chemotherapy have significantly impacted patient outcomes, increasing the rates of curative surgeries and reducing pelvic recurrence [1, 2].

Previous studies have demonstrated the importance of MRI for accurately assessing rectal cancer during baseline staging [3, 4]. MRI plays a crucial role in optimizing the selection of patients with no poor prognostic factors who are suitable for upfront surgery, as well as selecting those demonstrating poor prognostic factors who may benefit from neoadjuvant chemoradiotherapy (CRT) [5]. Furthermore, it enables response assessment after CRT by distinguishing between responding and nonresponding tumors and allowing for the selection of candidates for organ-preserving strategies [6]. Moreover, MRI aids in surgical planning, the evaluation of postoperative complications, and the detection of pelvic recurrence [7].

Improvements in treatment techniques provide a reduction in pelvic recurrence rates from over 30% to less than 10%, reaching around 5% in some cohorts [8]; however, it still remains an important posttreatment surveillance concern given the morbidity resulting in untreatable pain, fistulae formation, and reduced quality of life. Some recurrent lesions are prone to salvage surgical resection; therefore, the early detection of resectable lesions may impact patient prognosis [9].

Pelvic recurrence is defined as tumor recurrence in the pelvic structures of patients undergoing surgical treatment; this differs from the diagnosis in patients with a complete clinical response after CRT during follow-up (watch and wait), which is defined as “tumor regrowth”. Surveillance of patients after surgical treatment is routinely performed through physical examination, colonoscopy, which plays an important role in the early diagnosis of endoluminal recurrence, and computed tomography (CT), which is important for the diagnosis of distant metastases [10]. Therefore, given that MRI offers greater sensitivity than CT for detecting pelvic recurrence and hepatic metastases, MRI is essential for the diagnosis of extramural lesions (not detectable by colonoscopy) and for evaluating changes identified by CT [10].

Detection can be challenging during follow-up because neoplastic tissue can mimic postsurgical and postradiotherapy changes. Additionally, positron emission tomography with fluorodeoxyglucose (FDG-PET/CT) can assist in differentiating posttreatment changes from pelvic recurrence, providing higher sensitivity than CT, especially for distant metastases and lymph node involvement. However, FDG-PET/CT can also yield false positives (inflammatory activity) and has a lower sensitivity for small lesions and mucinous tumors [10, 11]. When considering resectable recurrent pelvic lesions, PET/CT can aid in the detection of metastatic diseases that may contraindicate surgery.

The resectability of pelvic recurrence is highly related to lesion location; therefore, reports should clearly describe the relevant anatomic relationships so that surgeons are able to successfully perform a clear-margin resection [12]. Pelvic recurrence patterns have significant prognostic implications and are associated with the potential for successful therapeutic strategies; therefore, radiologists play a key role in evaluating these patients.

This study aimed to review the main risk factors and imaging patterns of local pelvic recurrence, discuss the resectability criteria, and identify the relevant information required to define a better treatment strategy in multidisciplinary team discussions.

## Review on the surgical techniques and main imaging findings related to the increased risk of local recurrence

### Surgical techniques

Total mesorectal excision (TME) is the cornerstone surgical treatment for patients with rectal cancer [13]. During the procedure, the mesorectum—harboring the primary tumor, potentially metastatic lymph nodes, and vascular deposits within the mesorectal fat—is excised. In optimal surgical specimens, the circumferential resection margin (CRM) corresponds to the mesorectal fascia (MRF), and CRM positivity indicates an increased risk of local recurrence and poorer survival outcomes [14].

By respecting the outlines of the MRF and considering embryological principles, TME provides radical oncological resection, as well as reduces intra- and postoperative morbidity and mortality, while preserving vascular and nervous structures [15].

**Fig. 1:**
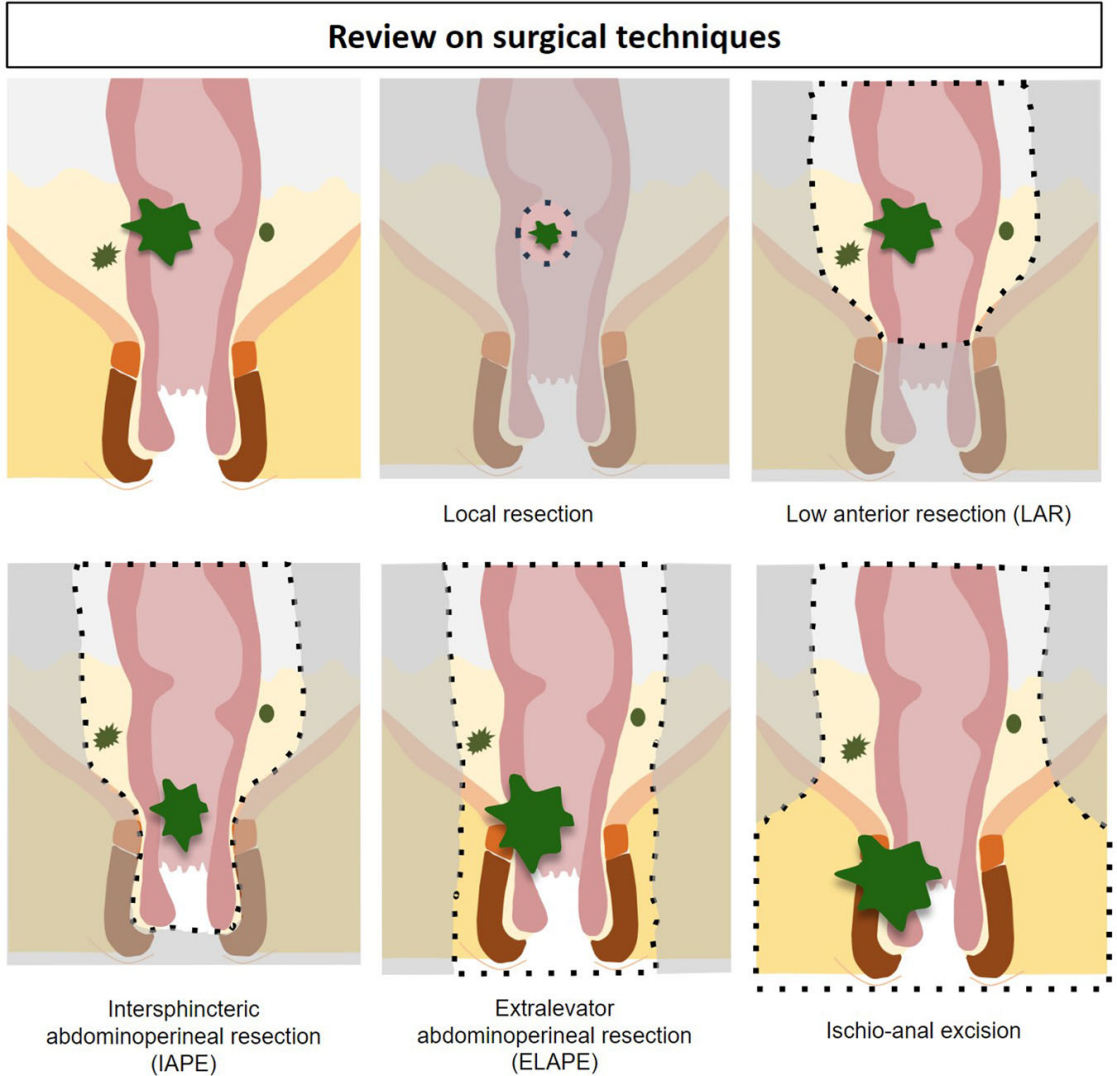
Review on surgical techniques

Familiarity with surgical techniques enables radiologists to identify expected findings on postoperative MRI evaluation. The most common surgical procedures adopted for rectal cancer treatment [16, 17] are (Fig. 1):

- Local resection: appropriate for early tumors (Tis/earlyT1).
- Low anterior resection (LAR): low-lying tumors above the intersphincteric plane. Anterior resection involves TME with colorectal anastomosis and is frequently associated with problematic intestinal dysfunction, characterized as “Low Anterior Resection Syndrome” (LARS), causing chronic pain, sexual dysfunction, and urinary and fecal incontinence. LAR syndrome manifests in 35% of patients after surgery alone; however, this rate increases significantly to 64% in patients treated with preoperative radiotherapy [16].
- Abdominoperineal resection (APR): lower rectal tumors and/or an unsafe intersphincteric plane. Patients require a permanent colostomy. APR has at least three different approaches:
   ∘ Intersphincteric abdominoperineal resection involves removing a tumor that invades part of the muscularis propria but does not penetrate through its full thickness.
   ∘ Extralevator abdominoperineal resection is performed when the tumor invades the full thickness of the muscularis propria or extends beyond the intersphincteric space.
   ∘ Ischioanal excision involves resecting wider margins using a surgical technique that shares similarities with the approach used for relapsed anal squamous cell carcinoma.

In addition to these main techniques, other information on surgical treatment is important to adequately interpret postoperative MRI scans of locally advanced tumors. Examples include surgical manipulation of the bladder, ureters, prostate, seminal vesicle, uterus, and ovaries, lymphadenectomy, complete or partial resection of the vagina with reconstruction using a myocutaneous flap [18].

Other important information includes whether the pelvis is filled with tissue (prosthesis or autologous/omental flap), sacrectomy, and the technique for perineal reconstruction (primary closure, myocutaneous flaps) to help differentiate the usual postsurgical changes from complications and neoplastic recurrence [18, 19].

Finally, a report of gross (macroscopically visible) disease remaining postsurgery, or the inability to achieve complete tumor excision with adequate surgical margins, allows for a focused and thorough analysis of areas with a higher likelihood of local recurrence (classified as R0: completely resected tumor with a negative margin; R1: microscopic residual tumor; or R2: macroscopic residual tumor) [18]. In some cases, these areas may be marked with surgical clips that can be identified during certain sequences of the study, thereby guiding the radiologist’s analysis.

### Quality of resection

Inadequate mesorectum excision can increase the risk of pelvic recurrence; therefore, it is crucial to evaluate the quality of the mesorectal excision prior to postoperative MRI assessment.

High-quality TME specimen was reported by pathologists according to the plane of resection using the “Macroscopic Assessment of Mesorectal Excision” classification. It describes the integrity of the mesorectum in the specimen and assesses the quality of the TME via visual inspection and the use of cross-sectional slices of the segment with the tumor [20].

- The mesorectal plane, previously referred to as a good or complete mesorectum, denotes an intact mesorectum with minor irregularities (< 5 mm).
- The intramesorectal plane, previously termed the moderate or moderately complete mesorectum, indicates a moderate bulk of the mesorectum with irregularities present on the mesorectal surface.
- The muscularis propria plane, previously described as a poor or incomplete mesorectum, refers to the minimal bulk of the mesorectum with defects extending down to the muscularis propria and/or a highly irregular circumferential resection margin, yet still reaching the muscularis propria.
- The intramuscular/submucosal plane denotes perforations or missing areas within the muscularis.

It is possible to recognize the residual mesorectum during postoperative follow-up MRI studies. Residual cranial fat tissue, at the level or below the anastomosis, is indicative of the residual mesorectum [21].

In addition to evaluating the quality of the TME, it is helpful to understand the surgical technique employed and expected changes (acute and chronic) in postoperative MRI assessment to improve the detection rates of local recurrence.

Radiologists should always compare the tumor height on baseline MRI with the plane of the anastomosis to assess whether the tumor has been resected with sufficient distal margins, and always check the pathological report (when available) to identify risk factors for pelvic recurrence [22].

### Risk factors for pelvic recurrence

Optimal surgical and neoadjuvant treatments aim to prevent local rectal cancer recurrence and reduce the risk of metastatic disease. However, some patients exhibit pelvic recurrence associated with multiple tumor-related features [23]. Although local recurrence is more frequent within 2 years of surgical resection of the primary tumor, late recurrences may occur [24]. The clinical manifestations can vary from asymptomatic, nonspecific symptoms, to pelvic pain, bleeding, and fistulas [24]. Synchronous metastatic disease occurs in approximately 50% of the patients diagnosed with local recurrence [25].

Treatment is complex, and MRI plays a crucial role in the decision-making process for surgical resection. However, in some cases, differentiating a locally recurrent lesion from benign tissue or fibrosis is challenging. Therefore, diffusion-weighted imaging (DWI) is helpful during follow-up assessment, and comparisons with previous studies may reveal growing lesions along the pelvis. Clinical assessments, including digital rectal examinations and colonoscopy, should also be considered [26].

As discussed below, pelvic recurrence is affected by multifactorial covariates [22, 23, 27–29] (Table 1):

**Table 1:** Poor prognostic factors

Low rectal tumor
Surgeries with a report of macroscopically visible disease or the inability to achieve complete excision of the tumor with adequate surgical margins
Positive mesorectal fascia on preoperative scans
High-grade tumor
Mucinous adenocarcinoma
Extramural vascular invasion
Involved lateral pelvic lymph nodes
Circumferential resection margin involvement
Poor response to neoadjuvant chemoradiotherapy
Anastomotic dehiscence
Tumor perforation during surgery
Specimen with incomplete mesorectum reported by pathologists
Advanced tumor (T3c, T3d, T4)

### Types of recurrence

Rectal cancer recurrence patterns have been categorized by pelvic compartments based on the involved anatomical pelvic region. As improvements in treatment have increased, the lateral and posterior forms (presacral) have become more common and have a poorer prognosis than anastomotic and anterior recurrence [12, 28].

Rectal cancer recurrence patterns are classified as follows:

- Axial: anastomosis (most common anterior or lower anastomosis, on the staple line), residual mesorectum, and perineal soft tissue;
- Posterior: presacral fascia, sacrum, coccyx, and nerve roots;
- Anterior: bladder, uterus, vagina, prostate, and seminal vesicle;
- Lateral: ureters, iliac vessels, pelvic lymph nodes, nerves, lateral wall musculature, and pelvic bones.

### How to perform postoperative MRI

The MRI protocol for the postoperative follow-up of rectal cancer includes T2-weighted fast spin-echo sequences acquired in the sagittal, axial, and coronal planes. Gadolinium-enhanced, fat-suppressed T1-weighted sequences and DWI can also be included. High-field-strength MRI (1.5 T or 3.0 T) is preferred for both staging and restaging, each of which has its advantages and disadvantages; some studies show that both exhibit similar accuracies if the imaging parameters are adjusted appropriately [30]. MRI 3.0 T can offer better spatial resolution owing to its increased signal-to-noise ratio; however, it may have disadvantages related to magnetic susceptibility artifacts caused by bowel gas, which may occur during DWI, a sequence that is particularly helpful for assessing residual tumors.

It is important to emphasize that bowel preparation is not necessary for rectal MRI. To decrease gas-induced susceptibility artifacts and ensure optimal image quality, especially in motion-sensitive sequences, patients should evacuate their rectal contents before acquisition. Some institutions recommend the use of microenemas before rectal MRI [31]; however, some services choose not to perform bowel preparation of any kind with satisfactory images. Endorectal filling is not routinely recommended due to compression of the mesorectal fat [32]. Although not mandatory, it is also recommended to use intravenously administered antispasmodic agents (e.g., butylscopolamine), which are helpful in reducing motion artifacts related to peristalsis of the rectum or adjacent bowel.

### How to assess local recurrence

In the assessment of postoperative follow-up MRI findings for rectal neoplasia, several details are important for proper interpretation (Table 2).

- Evaluation results of previous examinations, including digital rectal examination, rectoscopy, and baseline MRI.
- What was the location of the primary tumor?
- Are there any risk factors for pelvic recurrence?
- Did the patient complete neoadjuvant chemoradiotherapy course?
- Which type of surgery was performed?
- Was TME performed?
- What was the result of the pathology report? Were clear surgical margins achieved?
- What is the time interval between the surgery and the patient’s current examination? (Differentiate between acute and chronic postoperative changes).

**Table 2:**
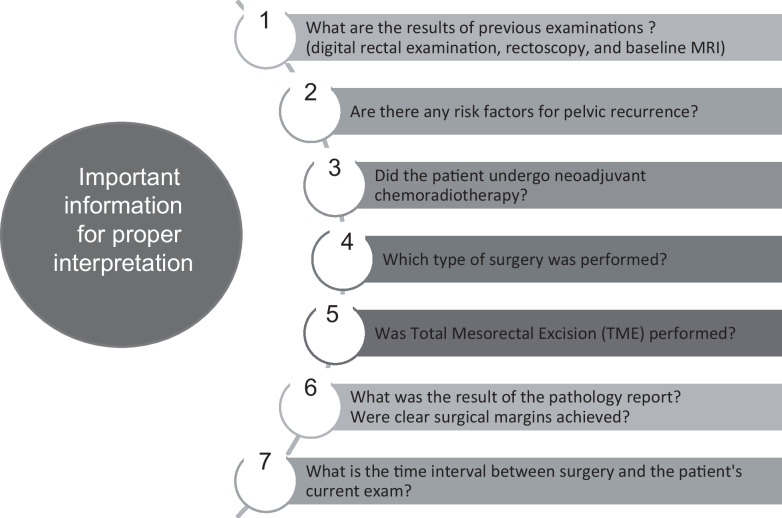
Important information for proper interpretation of postoperative follow-up MRI for rectal cancer

Middle and low rectal tumors were defined as those located below the peritoneal reflection, and at the level or below the anorectal ring, respectively. As the mesorectum tapers, the distance to the resection margin decreases, leading to an increased risk of margin positivity. The mesorectum is primarily drained by the mid and inferior rectal vessels, with a higher rate of spread to the lateral pelvic and inguinal lymph nodes. On the other hand, high rectal tumors located above the peritoneal reflection have a higher risk of perineal infiltration and metastasis to the superior rectal and inferior mesenteric lymph nodes [23, 33].

After assessing the initial staging, treatments performed, and risk factors for recurrence, it is important to systematically evaluate the MRI findings and describe the extent of neoplastic recurrence and the involved structures—which will ultimately guide surgical planning—in detail (Table 3).

1. Assessment of the axial compartment:
  First, the anastomotic topography should be evaluated regarding the location of the primary tumor to identify if the lesion was removed with sufficient margins (if available, the anatomopathological report of the surgical specimen should be reviewed). Nodular areas with intermediate T2 signal intensities should be considered in viable neoplastic tissues. Note that postoperative and radiation-induced injuries may make evaluation difficult; therefore, comparison with previous examinations is extremely important to identify new lesions and progressive growth.
2. Assessment of the anterior compartment:
  The presence of invasion into the anterior compartment structures should be determined (bladder, prostate/vesicle seminal, uterus/vagina).
3. Assessment of the posterior compartment:
  Whether the tumor invades the retrorectal space, presacral fascia, or sacral vertebra should be determined. The level of invasion should be described when sacral invasion occurs.
4. Assessment of the lateral compartment:
  It is important to recognize that early diagnosis of tumor growth along the fascia can be challenging. Whether there was invasion through the fascia into the lateral compartment; involvement of the ureters, internal iliac vessels, and external iliac vessels; bony invasion of the lateral pelvic sidewall; tumor extension into the sciatic nerve; or infiltration of the sacral nerve roots should be determined.
5. Assessment of the lower compartment:
  The presence of levator ani muscle infiltration, involvement of the ischioanal fossa, or periurethral or retropubic infiltration should be determined.
6. Assessment of postoperative changes:
  Screening for edema/fluid collections, signs of anastomotic dehiscence, cystic formations, or any other changes that may mimic recurrence should be considered in a follow-up study. PET/CT and biopsy may help resolve diagnostic uncertainties.

**Table 3:** Systematically evaluate MRI

Assessment of the axial compartment	Anastomosis topography in relation to the location of the primary tumor (sufficient surgical margins). Look for nodular areas with intermediate T2 signal intensity of viable neoplastic tissue. Remember that postoperative and actinic changes may make evaluation difficult, so it is extremely important to compare with previous exams to identify new lesions and progressive growth. Determine if there is tumor extension above peritoneal reflection space Involvement of lymph nodes.
Assessment of the anterior compartment	Determine if there is invasion into anterior compartment structures (bladder, prostate/vesicle seminal, uterus/vagina)
Assessment of the posterior compartment	Determine if the tumor invades the retrorectal space, presacral fascia, or sacral vertebra. Whenever there is sacral invasion, describe the level of invasion.
Assessment of the lateral compartment	Determine if there is invasion through the fascia into the lateral compartment, involvement of ureters, internal iliac vessels, external iliac vessels, bony invasion of the lateral pelvic sidewall, tumor extending into the sciatic nerve, or infiltration of sacral nerve roots.
Assessment of the lower compartment	Determine if there is levator muscle infiltration, involvement of the ischioanal fossa, or periurethral or retropubic infiltration.
Assessment of postoperative changes	Look for edema/fluid collections Signs of anastomotic dehiscence Cystic formations Any other changes that may mimic recurrence and should raise attention in the follow-up study. PET-CT and biopsy may help with diagnostic uncertainties.

## MRI patterns of local recurrence

### Axial

Anastomotic pelvic recurrence is relatively frequent, especially in nonirradiated, anterior, and low-resection cases, which have higher rates of compromised margins and MRF involvement [28]. Postoperative MRI changes such as architectural distortion, staplers in anastomotic regions, fibrotic scar tissues, treatment-related changes, and inflammatory lesions make it challenging to differentiate viable neoplastic tissues [26].

Anastomotic recurrence can be classified as intraluminal, extraluminal, or combined. Intraluminal recurrence, usually at the anastomotic line, is less common and better visualized in endoscopic studies [34, 35].

**Fig. 2:**
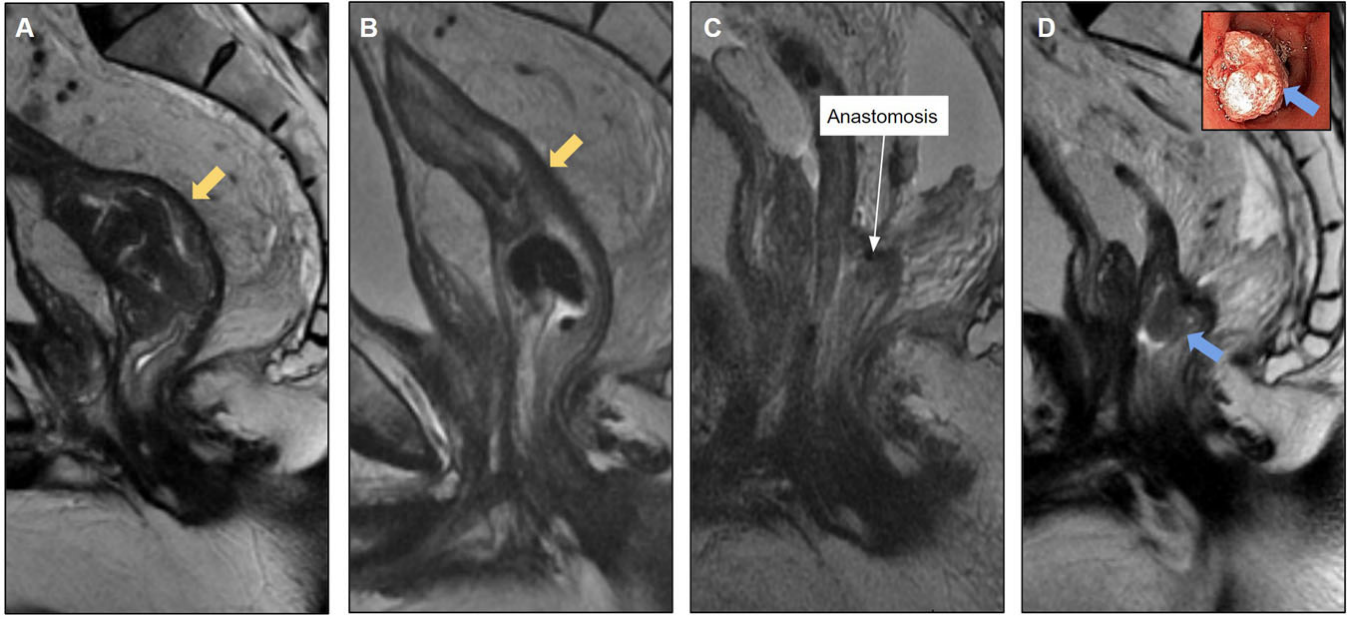
Case of anastomotic recurrence. **A** Sagittal T2-weighted image: baseline mrT2N1 (yellow arrow), mesorectal fascia (MRF) clear and no extramural vascular invasion (EMVI). **B** Post-long-course chemoradiotherapy (CRT) restaging shows response tumor regression grade (TRG) 2 (yellow arrow). **C** Follow-up after mesorectal excision (ypT2N0), with clear circumferential resection margin (5 mm) and insufficient distal margin: 1.6 cm. **D** Four months after surgery. Colorectal anastomosis recurrence was detected by endoscopic examination (blue arrow). MRI to plan surgical resection. Absence of sacral, neural, lateral wall and other adjacent organs invasion. Rectosigmoidectomy for anastomotic recurrence resection was proposed

MRI is important for anastomotic evaluation because extramural lesions may not be accessible by colonoscopy. Figure 2 shows pre- and postoperative images of a rectal tumor resected with an insufficient distal margin, with anastomosis recurrence characterized by growing solid lesions not seen on the previous postoperative MRI.

**Fig. 3:**
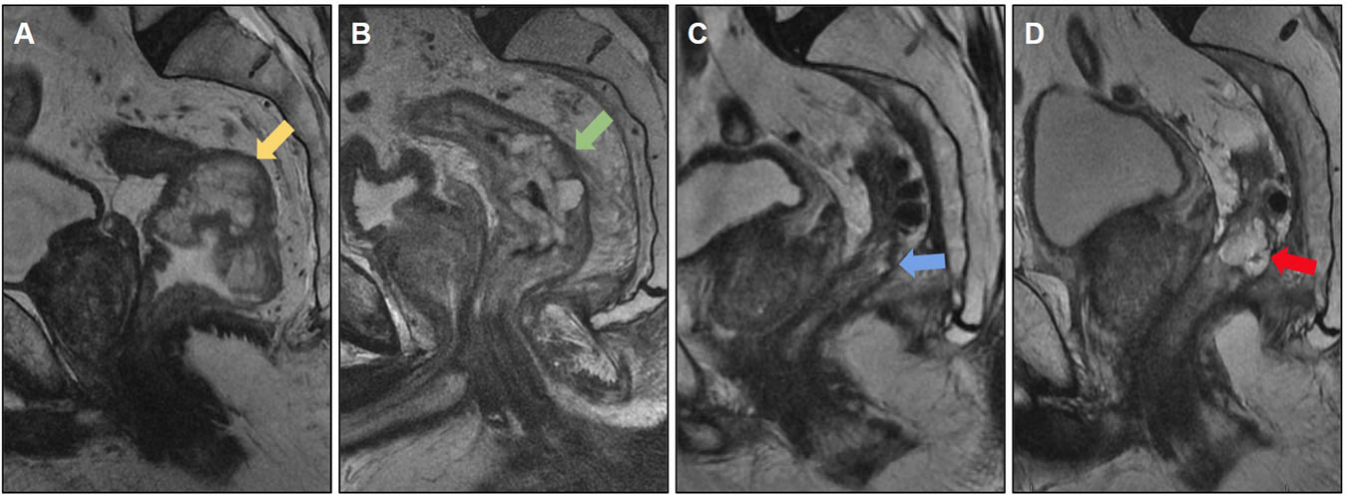
**A** Baseline MRI showing mucinous adenocarcinoma, mrT2N2 stage, negative mesorectal fascia (MRF), and negative extramural vascular invasion (EMVI) (yellow arrow). **B** MRI 8 weeks after neoadjuvant chemoradiotherapy (CRT) displaying poor response with tumor regression grade (TRG 5) (green arrow). **C** MRI 6 months after rectosigmoidectomy revealing incomplete mesorectal resection and insufficient distal margin (0.7 cm). The colorectal anastomosis exhibits a discrete area with a high T2 signal, casting doubt on postoperative changes or mucin (blue arrow). **D** MRI 1.5 years post-surgery demonstrating recurrence at the anastomosis site. Tissue growth with a high T2 signal was suggestive of recurrent tumor (red arrow). Subsequent surgical planning involved posterior abdominoperineal resection (APR)

Figure 3 shows another example of recurrence in the anastomosis of a mucinous adenocarcinoma with a poor CRT response.

Radiologists must carefully inspect the anastomosis, identify suspicious solid tissues, and always compare them with previous examinations, if available, allowing for the detection of small growing lesions [36]. In such cases, DWI helps detect tumor recurrence [37]. Intravenous contrast agent is not required, although it may aid in the detection of recurrence when mucinous lesions are considered [27]. Additionally, the anastomotic location of rectal cancer recurrence is a key factor in assessing resectability. For surgical planning, it is important to describe the distance between the lesion and anal verge, and whether there is infiltration of the levator ani muscle. Therefore, check for endoscopy or digital rectal examination; and map lesions to plan surgical resection.

### Lateral

Lateral pelvic lymph nodes represent a risk factor for lateral pelvic recurrence, especially in patients with low rectal cancer that has two lymphatic outflows: the mesenteric lymphatic drainage and internal iliac/obturator vessels [38]. These tumors are not routinely resected during TME or APR in Western countries. In addition to increasing the surgical time and risk of bleeding, radiation-induced injury can complicate resection and increase the risk of injury to the inferior hypogastric plexus [39, 40].

Internal iliac and obturator lymph nodes with a short axis ≥ 7 mm at initial staging are at increased risk for metastatic involvement [41, 42]. In the evaluation after neoadjuvant CRT, the value considered suspicious is > 4 mm on the short axis, and a multidisciplinary evaluation should be performed to evaluate surgical resection, as several studies have demonstrated a reduction in the rate of pelvic recurrence with lymphadenectomy of suspected lymph nodes. By contrast, Japanese studies have demonstrated that involvement of the external iliac lymph nodes is a risk factor for distant metastases, and not for pelvic recurrence [43, 44].

Komori et al [45] demonstrated a 7.3% rate of anatomopathological diagnosis for metastatic involvement of routinely resected lymph nodes without suspicious morphological changes (short axis diameter on CT < 10 mm). Furthermore, in the analysis of pathological lateral pelvic lymph node metastasis, 21.4% of the lymph nodes had a short axis diameter of 5–10 mm, while 5.2% had a diameter of < 5 mm. Thus, the study demonstrated that a cutoff of 5 mm is an important predictive factor for lateral pelvic lymph node metastasis; the 5 mm cutoff is even more relevant for patients who will not receive neoadjuvant CRT.

**Fig. 4:**
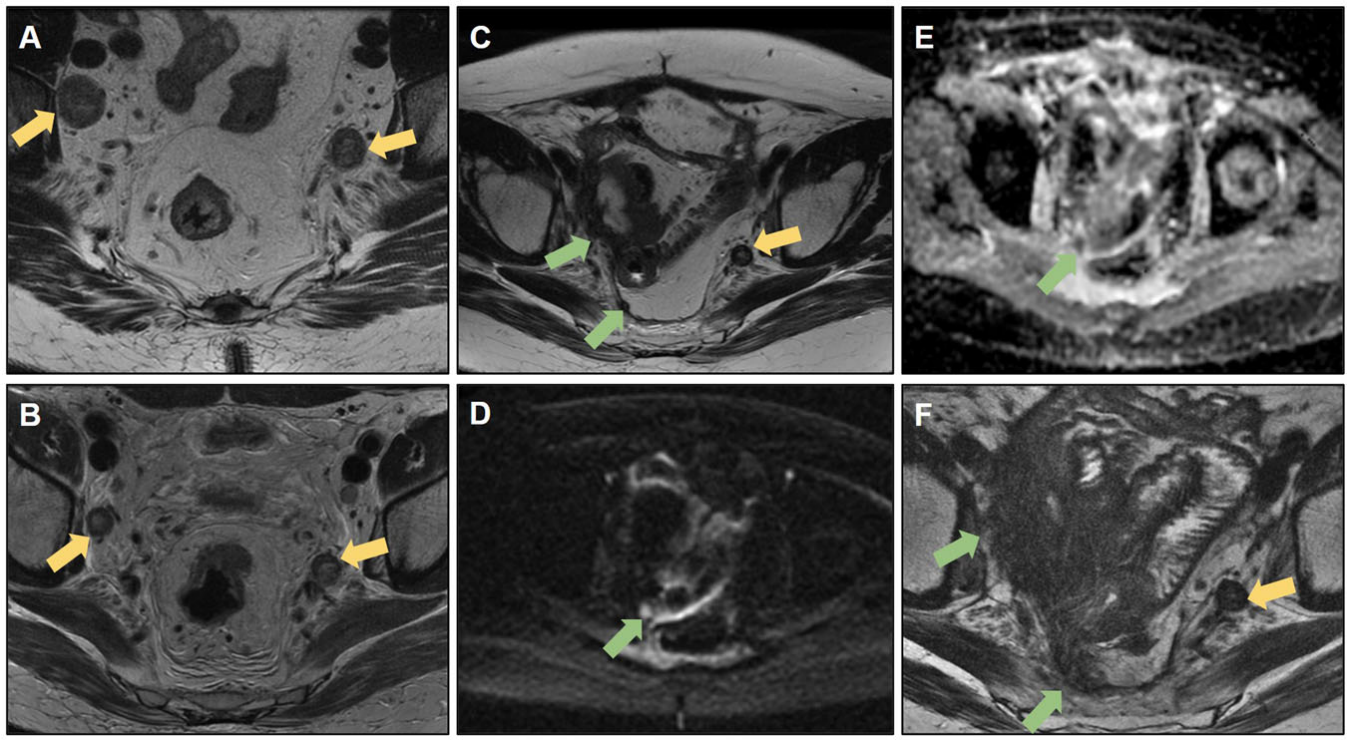
**A** Baseline MRI showing mrT4N1 status, with infiltration of the levator ani muscle, positive extramural vascular invasion (EMVI), involvement of the mesorectal fascia (MRF), and intersphincteric plane, with suspicious bilateral pelvic lateral lymph nodes (yellow arrow). **B** MRI restaging after neoadjuvant chemoradiotherapy (CRT) with a poor response (mrTRG 4), demonstrating reduction in the size of the pelvic lateral lymph nodes (yellow arrow). **C** Patient underwent abdominoperineal resection (APR) and bilateral pelvic lymphadenectomy; however, the persistence of a suspicious left pelvic lateral lymph node is noted (yellow arrow). Suspicious poorly defined tissue suggestive of recurrence is evident in the right pelvic sidewall (green arrow). **D** and **E** show diffusion restriction with a high signal on diffusion-weighted imaging (DWI), and a low signal on ADC of the peritoneal implant (green arrow). **F** Follow-up MRI revealing unresectable pelvic sidewall recurrence 1 year after surgery, with involvement of the peritoneum, bladder, seminal vesicles, and presacral space (green arrow)

Figure 4 shows an example of bilateral involved obturator lymph nodes on baseline MRI that underwent lymphadenectomy. The iliac lymph node on the left side was not resected owing to the risk of intraoperative bleeding. A follow-up assessment revealed an infiltrative lesion involving the iliac vessels on the right side. Muscle and bone involvement, as well as the distance of the tumor from these structures, must be reported. Lateral pelvic recurrence can extend to the anterior and posterior structures and must be carefully staged to assess resectability.

**Fig. 5:**
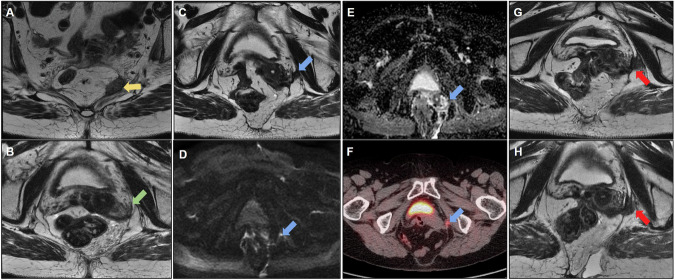
**A** Follow-up of a patient who underwent rectosigmoidectomy with coloanal anastomosis, revealing recurrence in the pelvic sidewall (yellow arrow). **B** The patient underwent abdominoperineal amputation with resection of the recurrent pelvic sidewall, showing usual postoperative changes (green arrow). **C** During follow-up, there was a progressive increase in serum levels of the tumor marker (CEA), and a nodular formation with intermediate signal intensity on T2-weighted imaging was visualized in the left pelvic sidewall (blue arrow), with diffusion restriction with a high signal on diffusion-weighted imaging (DWI) (**D**), and a low signal on ADC (**E**). **F** Positive fluorodeoxyglucose (FDG) uptake on positron emission tomography (PET/CT) (blue arrow), suspicious for neoplastic recurrence. **G**, **H** Follow-up MRI demonstrating growth of the neoplastic tissue (red arrow)

Figure 5 shows resected left lateral pelvic recurrence, evolving with another focus on the recurrent lesion on the left pelvic lateral wall.

**Fig. 6:**
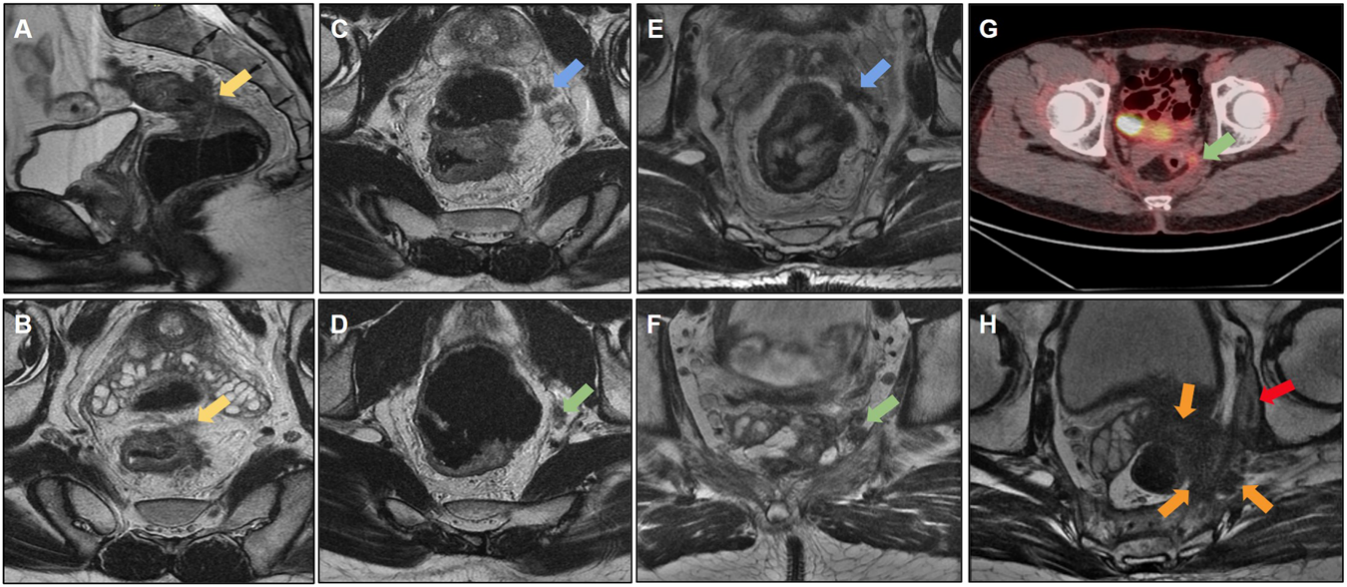
**A**, **B** Sagittal and axial T2-weighted image: baseline mrT4aN1c (yellow arrow), mesorectal fascia (MRF) positive and extramural vascular invasion positive (EMVI). **C** Tumor deposit in the rectoprostatic recess (blue arrow) and (**D**) suspicious lateral pelvic lymph node on the left (green arrow) were also characterized. **E** Post-long-course chemoradiotherapy (CRT) restaging demonstrates a response tumor regression grade (TRG) 3, with the persistence of the tumor deposit (blue arrow) and pelvic lateral lymph node on the left (not included in the image). **F** Follow-up after mesorectal excision with colorectal anastomosis revealed the presence of poorly defined tissue with intermediate signal intensity on T2-weighted imaging in the area where the previous tumor deposit and lateral pelvic lymph node were identified, raising suspicion regarding neoplastic recurrence (green arrow). **G** Positron emission tomography (PET)-computed tomography (CT) scan demonstrating increased radiopharmaceutical uptake in the region adjacent to the left colorectal anastomosis, corresponding to the suspicious tissue (green arrow). **H** At the 2-year follow-up, there was a notable progression of neoplastic tissue, characterized by infiltration of the rectum, prostate, left seminal vesicle, and pelvic lateral wall muscles (orange arrow). Notably, edema of the left internal obturator muscle is observed, likely related to denervation (red arrow)

Figure 6 shows an example of a tumor deposit in the left rectoprostatic space that was not resected but evolved into an extensive unresectable lesion during follow-up.

Therefore, careful evaluation of the pelvic lateral lymph nodes is necessary in the initial staging, as well as in the follow-up of these patients, for early diagnosis to avoid progression and enable surgical resection [25].

### Posterior

Posterior recurrence is characterized by involvement of the presacral fascia, sacrum, coccyx, and nerve roots. Presacral local recurrence is the most common type of local recurrence and has a poor prognosis [28].

Locally recurrent tissues usually have a higher signal intensity than muscle on T2-weighted imaging. However, granulation tissue, hematoma, and radiation-induced inflammatory changes are indistinguishable from tumor recurrence [46]. Thus, attention is required for focal heterogeneous lesions, marked contrast enhancement, early invasive behavior, and asymmetric appearance, which are suspicious for local recurrence [28, 47, 48].

Furthermore, an increase in lesion size over time on serial follow-up imaging is helpful in differentiating postoperative and postradiation therapy changes from tumor recurrence [25].

When staging posterior recurrence, it is important to describe the involvement of the retrorectal space, presacral fascia/space, sacral bone level, and infiltration of the sacral nerve roots (irregular thickening, increased T2 signal, enhancement, diffusion restriction) and muscles (signs of chronic muscle denervation: atrophy and fat replacement; acute changes: edema) [49, 50].

**Fig. 7:**
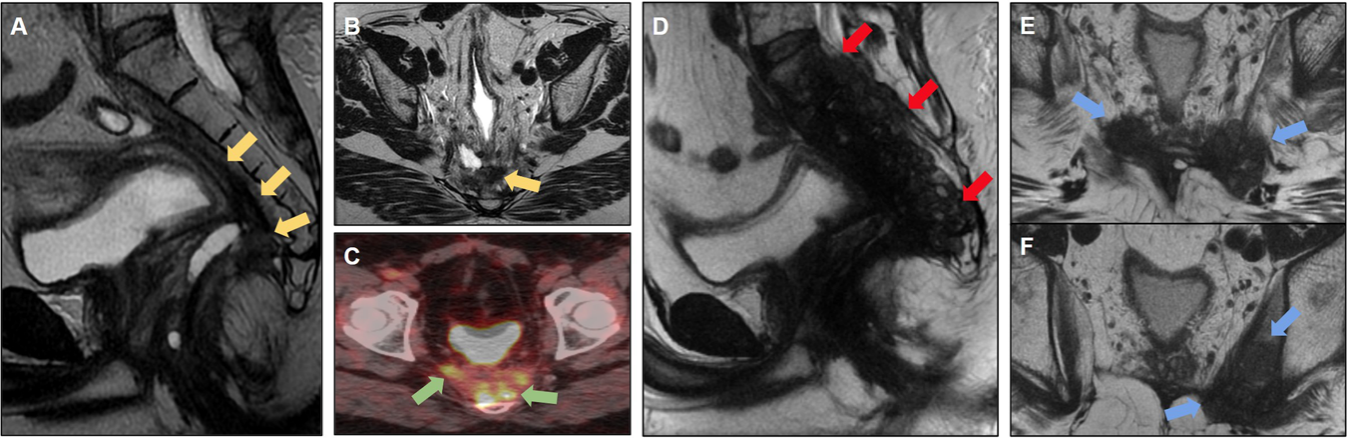
**A** Sagittal and (**B**) axial T2-weighted images; the patient underwent abdominoperineal amputation for low rectal cancer with positive circumferential resection margin, developing poorly defined tissue in the posterior pelvic space (yellow arrows), suspicious for neoplastic recurrence. **C** Positron emission tomography (PET)-computed tomography (CT) demonstrating increased glycolytic metabolism (fluorodeoxyglucose (FDG)) in the presacral tissue and internal iliac arteries, consistent with neoplastic disease (green arrows). No distant metastasis was observed; however, surgery was contraindicated due to the patient’s clinical condition. **D** Follow-up evidence of an unresectable lesion infiltrating the sacrum and invading the bilateral superior sacral foramen, left lumbosacral plexus, and spinal canal. The lesion extends into the presacral space, causing retraction of the bladder and internal iliac vessels to the left (red arrows). **E** and **F** also show extension into the lateral pelvic spaces (blue arrows)

Even in the absence of bone infiltration, the level of presacral fascial infiltration—which may contraindicate surgical treatment—must be described [25]. Figure 7 shows a case of posterior recurrence with an extensive lesion affecting the sacrum and extending bilaterally to the lateral wall.

### Anterior

Anterior recurrence is the least common type of recurrence, and its prognosis is generally better than presacral and lateral recurrence [28]. It is important to evaluate the extent of tumor recurrence involving the bladder, uterus, vagina, prostate, and seminal vesicles for adequate surgical planning; moreover, extended radical resection is often required to optimize the likelihood of achieving a tumor-free resection margin [51].

### Imaging pitfalls

**Fig. 8:**
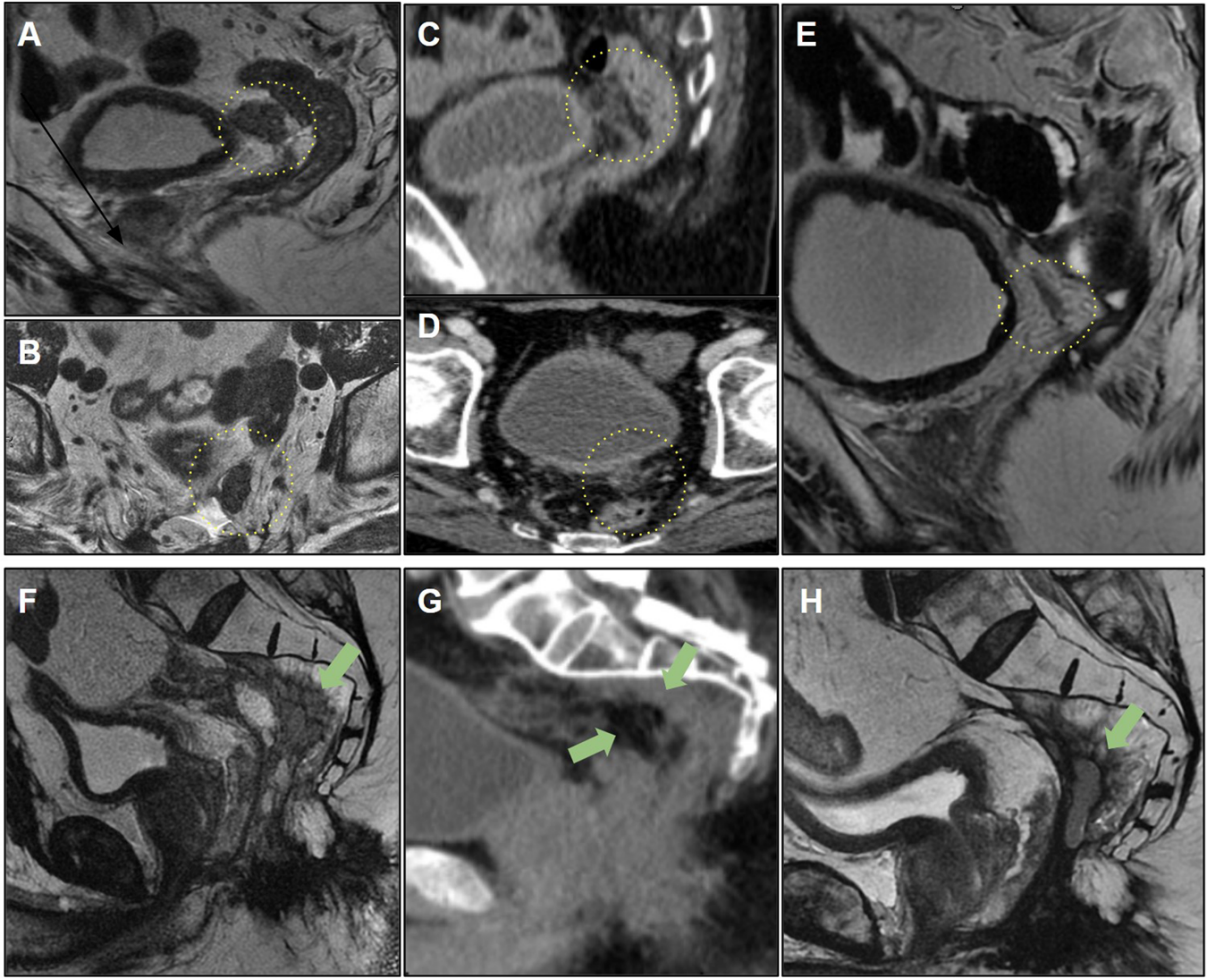
**A**, **B** The patient underwent rectosigmoidectomy (pT2N0). MRI 9 months after surgery revealed a nodular lesion with intermediate T2 signal anterior to the mesocolon (yellow circle). Suspected tumor recurrence. **C**, **D** Computed tomography (CT) demonstrating a fatty nodular lesion consistent with steatonecrosis (yellow circle). **E** Steatonecrosis focus reduced after 4 years of follow-up, and the absence of pelvic recurrence was observed. **F** Another case of a patient who underwent abdominoperineal amputation with omental flaps. Solid tissue on the surgical resection bed was misinterpreted as recurrence (green arrow). **G** Comparison of CT scans shows omental flaps mimicking pelvic recurrence (green arrow). **H** Follow-up examinations reveal tissue shrinkage with increased fibrotic content. Stability was observed for 5 years, supporting no signs of recurrence and indicating a pattern of postsurgical changes (green arrow)

Figure 8 shows some MRI patterns that may mimic neoplastic lesions that make the early diagnosis of recurrence challenging. Overdiagnosis may lead to unnecessary procedures; thus, it is important for radiologists to familiarize themselves with these imaging aspects to facilitate the evaluation of follow-up images. For example, areas of steatonecrosis can appear as viable neoplastic tissue on MRI; CT can aid the diagnosis, as the detection of macroscopic fat components is straightforward on CT.

**Fig. 9:**
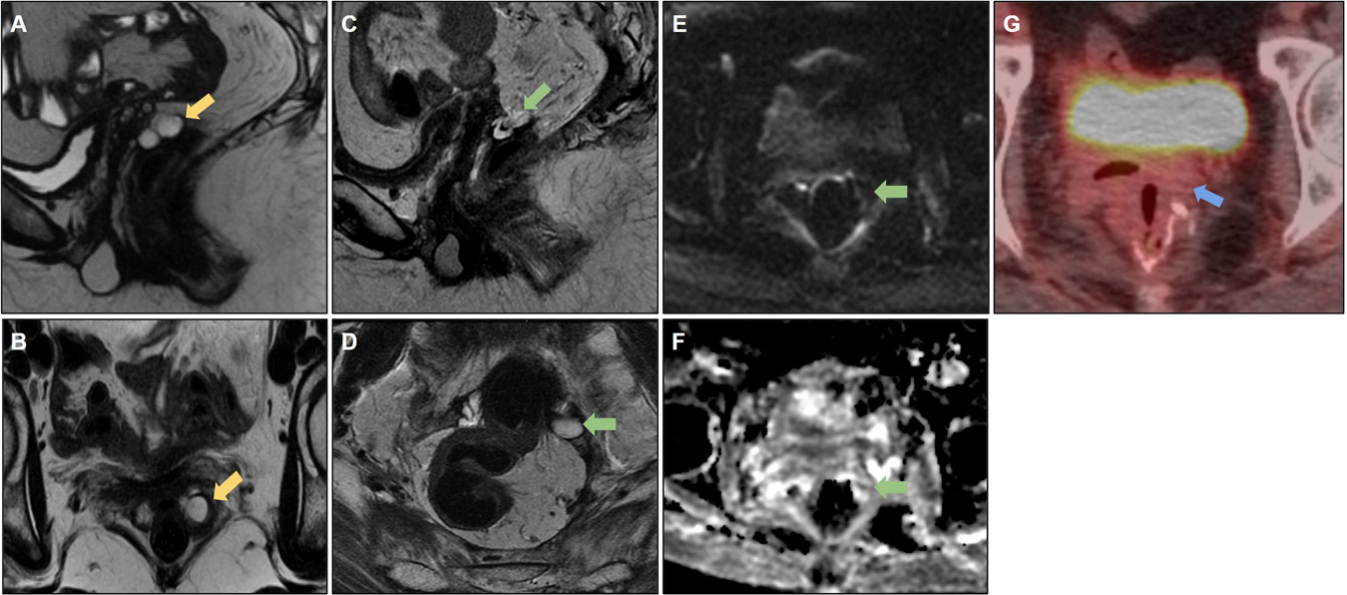
**A** Sagittal and **B** axial T2 following adjuvant chemoradiotherapy (CRT). A cystic formation was observed at the anastomosis site with peripheral intermediate T2 signal, raising suspicion for residual tissue/recurrence. The high T2 signal could potentially correspond to mucin. **C**, **D** MRI at the 6-month follow-up revealed persistent cystic formation, which remained stable with no evidence of growth. Diffusion-weighted imaging (DWI) (**E**) and ADC (**F**) do not exhibit any diffusion restrictions. **G** Positron emission tomography (PET)-computed tomography (CT) showing negative fluorodeoxyglucose (FDG) uptake at the location of cyst formation. Cystic formation was interpreted as a postsurgical change

Awareness of the surgical technique performed is also helpful; for example, omental flaps, which are used to decrease morbidity associated with reducing the size of the remaining dead space with well-vascularized tissue after APR, can also simulate recurrence [52]. Another surgical technique is the double-stapling technique, which is routinely used for anastomosis during TME. This technique has been sporadically associated with implantation cysts (Fig. 9) and should be considered in the differential diagnosis of cystic lesions and local recurrence [53]. Differentiation may be more difficult in cases of mucinous adenocarcinoma, and stable behavior on follow-up scans suggests the benign nature of this condition.

**Fig. 10:**
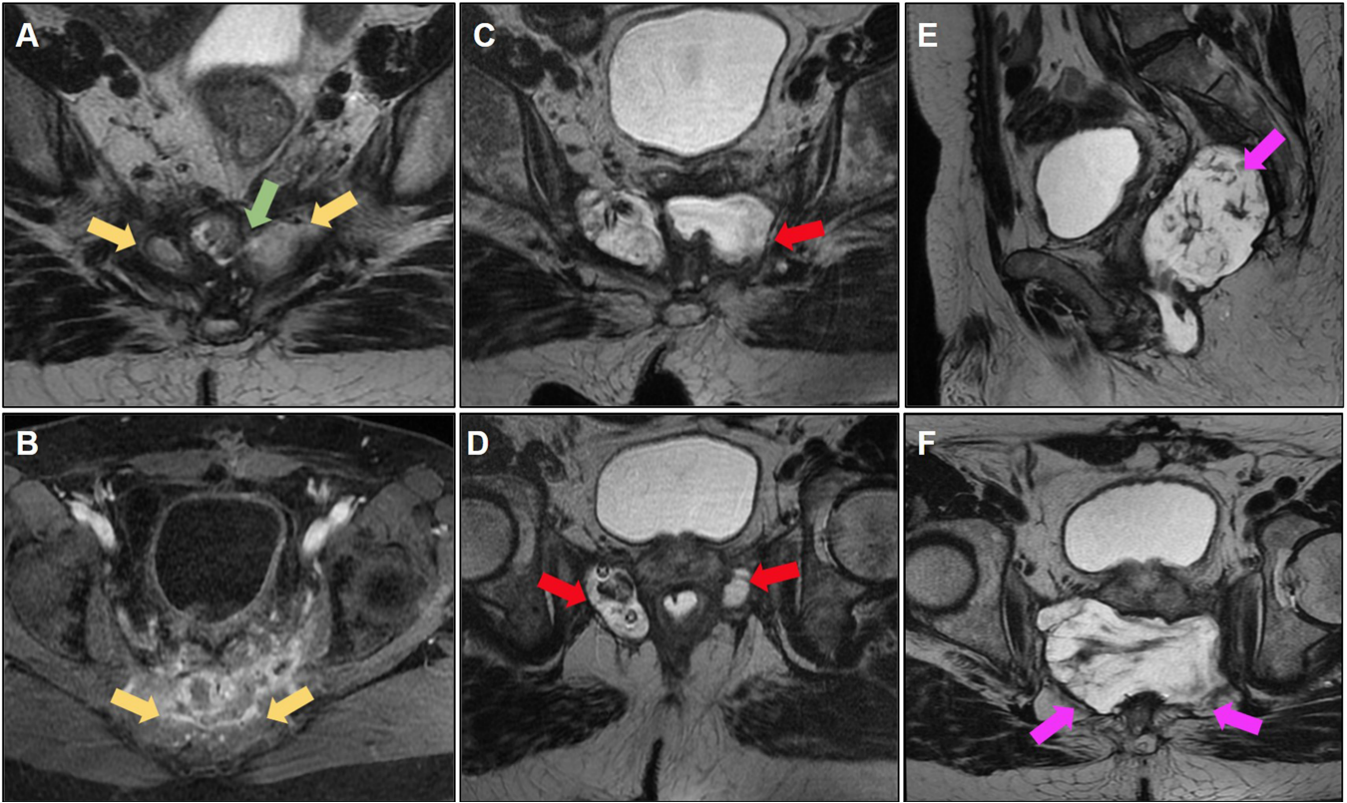
**A** A patient with mucinous adenocarcinoma of the rectum treated with surgery and radiotherapy underwent rectosigmoidectomy. They developed an extensive collection in the presacral space (yellow arrow) extending into the ischioanal fossae in the late postoperative period, associated with multiple fistulous tracts involving the colorectal and perianal anastomosis. **B** Axial T1 postcontrast image demonstrating collection in the presacral space (yellow arrow). **C** and **D** show enlargement of the collection, even after drainage (red arrow). **E**, **F** The patient underwent resection of the rectal stump with terminal colostomy, with persistence of the anal canal and diagnosis of mucinous recurrence in the surgical specimen, evolving with recurrence of the pelvic collection with high signal intensity on T2-weighted imaging and solid enhancing areas, suspicious for mucinous neoplastic recurrence

Figure 10 shows another postoperative case of mucinous adenocarcinoma that evolved with extensive collection in the presacral space extending to the ischioanal fossa, and was associated with multiple fistulous tracts with colorectal and perianal anastomoses. After surgical resection of the rectal stump and the diagnosis of mucinous recurrence, the patient ended up with a recurrence of collections with a mucinous component. It should be noted that it can be difficult to differentiate fluid collection from recurrence; in this case, solid areas with enhancement aided in the diagnosis of recurrence [54].

**Fig. 11:**
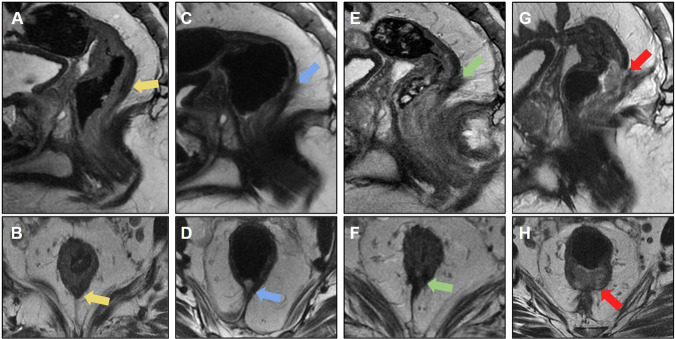
**A** Sagittal and **B** axial T2-weighted images. A patient with distal rectal cancer underwent local resection (pT2). MRI revealed a low signal intensity scar (yellow arrow) with no evidence of mesorectal disease. **C**, **D** Follow-up MRI at 5 months revealed missed changes within the mesorectal fat posterior to the scar (blue arrow). **E**, **F** Follow-up MRI at 1 year demonstrating missed scar recurrence extending along the mesorectal fat (green arrow). Changes from fat stranding to a nodular lesion along the mesorectal fat near the scar should raise suspicion of recurrence after local resection. Endoscopic studies failed to detect extraluminal recurrence. **G**, **H** Posterior follow-up at 1 year and 6 months revealed a mucosal lesion (red arrow). Local recurrence was detected using endoscopy with a positive mesorectal fascia. Treatment with chemoradiotherapy and total mesorectal excision was indicated

Figure 11 depicts a case of local resection of a rectal tumor with extrinsic growth of a small nodular area on the posterior wall along the scar of the resection bed. This case reinforces the importance of comparison with previous examinations for the early diagnosis of recurrent neoplastic tissue. Imaging techniques, such as endoscopic examination, CT, and PET-CT, can aid in early recurrence detection; however, according to some authors [54], PET-CT should be avoided for 6 months following pelvic radiation therapy to avoid incorrect interpretation, as inflammatory changes following radiation therapy can also affect PET findings. FDG-PET also performs poorly when evaluating recurrent, mucin-filled tumors [53] (Table 4).

**Table 4:** Differentiating pelvic recurrence from other benign lesions

Injury classification	T2-weighted image MRI	Dynamic contrast-enhanced (DCE) image MRI	Diffusion-weighted imaging (DWI)	Follow-up
Local recurrence tissues	Higher signal intensity than muscle on T2-weighted image MRI and asymmetric appearance	Marked contrast enhancement	Observed diffusion restriction (DWI/ADC)	Early invasive behavior
			
Other benign lesions	Low signal intensity on T2-weighted image MRI	Absence of notable contrast enhancement (T2 WI/DCE)	No evident restriction of diffusion is detected (T2 WI/ADC)	Stability of changes
			

### Resectability and unresectability criteria

Patients with pelvic recurrence have worse prognoses, with an impact on morbidity and mortality. The involvement of the sacrum and/or sacral nerves may cause sacral nerve pain, perineal ulcers, fistulas, bleeding, bowel and/or urinary tract obstruction, and sepsis [55]. These conditions are difficult to treat, and chemotherapy provides minimal benefits [56].

Surgical treatment may involve total pelvic exenteration and/or distal sacrectomy, and should only be considered in carefully selected populations [12]. This surgery can provide pain control, prolong survival, and possibly cure the disease [57]; however, careful systemic staging is recommended whenever local recurrence is diagnosed as it is a risk factor for metastatic disease [58].

It is important to describe in the report all the anatomic landmarks to guide the surgical team in evaluating potentially resectable lesions and helping in surgical planning. The suspicion of ureteric involvement is important for surgeons, and the involvement of the levator muscles or other pelvic floor structures does not preclude resection [25].

**Fig. 12:**
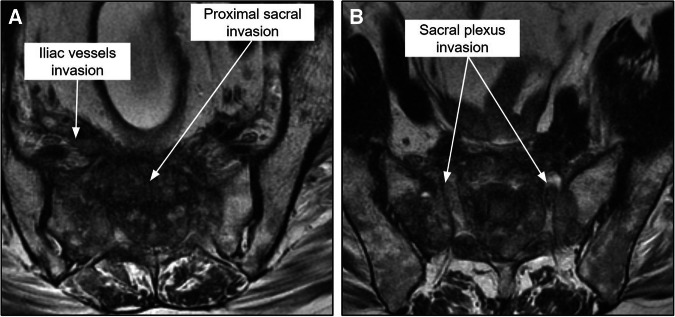
**A**, **B** Axial T2 images demonstrating unresectable posterior pelvic recurrence, with extensive involvement of the presacral space, sacrum (up to the level of S1), sacral plexus invasion, and bilateral iliac vessel invasion

The main MRI-determined unresectable lesions that contraindicate surgical treatment for pelvic recurrence are [12, 59, 60] (Fig. 12):

A. Proximal sacral invasion (S1/S2): proximal lesion resection leads to sacral instability.
B. Invasion of the nerve roots of the sacral plexus.
C. Tumor encasement of bilateral iliac vessels.
D. Extension of tumor into the sciatic notch.
E. Extensive pelvic sidewall involvement.

## Conclusion

The diagnosis of pelvic recurrence of rectal cancer remains challenging and has a significant impact on patient mortality and morbidity. MRI plays a crucial role in posttreatment follow-up, whether by identifying viable neoplastic tissues or acting as a tool for therapeutic planning and assessing the resectability of these lesions. However, postsurgical inflammatory changes related to radiotherapy and fibrosis make it difficult to detect initial lesions. This study aimed to review the main imaging patterns of pelvic recurrence and their implications for the surgical decision-making process. We therefore emphasize the importance of correlating MRI findings with those of other imaging methods—such as CT, PET, colonoscopy, and clinical examination—for proper evaluation and mapping of recurrences, with the aim of attempting curative surgery for recurrent rectal cancer.

## Abbreviations

APR: Abdominoperineal resection
CRM: Circumferential resection margin
CRT: Chemoradiotherapy
CT: Computed tomography
DCE: Dynamic contrast-enhanced
DWI: Diffusion-weighted imaging
EMVI: Extramural vascular invasion
FDG: Fluorine-18-deoxyglucose
LAR: Low anterior resection
MRF: Mesorectal fascia
MRI: Magnetic resonance imaging
PET: Positron emission tomography
TME: Total mesorectal excision

## References

[1] Taylor FGM, Swift RI, Blomqvist L, Brown G (2008) A systematic approach to the interpretation of preoperative staging MRI for rectal cancer. AJR Am J Roentgenol 191:1827–1835. 10.2214/AJR.08.1004 19020255 10.2214/AJR.08.1004

[2] Wang J, Prabhakaran S, Larach T et al (2022) Treatment strategies for locally recurrent rectal cancer. Eur J Surg Oncol 48:2292–2298. 10.1016/j.ejso.2022.05.011 35637097 10.1016/j.ejso.2022.05.011

[3] Brown G, Davies S, Williams GT et al (2004) Effectiveness of preoperative staging in rectal cancer: digital rectal examination, endoluminal ultrasound or magnetic resonance imaging? Br J Cancer 91:23–29. 10.1038/sj.bjc.6601871 15188013 10.1038/sj.bjc.6601871 PMC2364763

[4] MERCURY Study Group (2006) Diagnostic accuracy of preoperative magnetic resonance imaging in predicting curative resection of rectal cancer: prospective observational study. BMJ 333:779. 10.1136/bmj.38937.646400.55 16984925 10.1136/bmj.38937.646400.55 PMC1602032

[5] Patel UB, Taylor F, Blomqvist L et al (2011) Magnetic resonance imaging–detected tumor response for locally advanced rectal cancer predicts survival outcomes: MERCURY experience. J Clin Oncol 29:3753–3760. 10.1200/JCO.2011.34.9068 21876084 10.1200/JCO.2011.34.9068

[6] Smith JJ, Strombom P, Chow OS et al (2019) Assessment of a watch-and-wait strategy for rectal cancer in patients with a complete response after neoadjuvant therapy. JAMA Oncol 5:e185896. 10.1001/jamaoncol.2018.5896 30629084 10.1001/jamaoncol.2018.5896 PMC6459120

[7] Bhoday J, Balyasnikova S, Wale A, Brown G (2017) How should imaging direct/orient management of rectal cancer? Clin Colon Rectal Surg 30:297–312. 10.1055/s-0037-1606107 10.1055/s-0037-1606107 PMC5703668 29184465

[8] Jootun N, Sengupta S, Cunningham C et al (2020) Neoadjuvant radiotherapy in rectal cancer—less is more? Colorectal Dis 22:261–268. 10.1111/codi.14863 31556218 10.1111/codi.14863

[9] Palmer G, Martling A, Cedermark B, Holm T (2007) A population-based study on the management and outcome in patients with locally recurrent rectal cancer. Ann Surg Oncol 14:447–454 17139457 10.1245/s10434-006-9256-9

[10] Young Patrick E, Womeldorph CM, Johnson EK et al (2014) Early detection of colorectal cancer recurrence in patients undergoing surgery with curative intent: current status and challenges. J Cancer 5:262–271. 10.7150/jca.7988 24790654 10.7150/jca.7988 PMC3982039

[11] Inoue A, Sheedy SP, Wells ML et al (2023) Rectal cancer pelvic recurrence: imaging patterns and key concepts to guide treatment planning. Abdom Radiol (NY) 48:1867–1879. 10.1007/s00261-022-03746-4 36737522 10.1007/s00261-022-03746-4 PMC12285713

[12] Colosio A, Fornès P, Soyer P, Lewin M, Loock M, Hoeffel C (2013) Local colorectal cancer recurrence: pelvic MRI evaluation. Abdom Imaging 38:72–81. 10.1007/s00261-012-9891-5 22484342 10.1007/s00261-012-9891-5

[13] Komen N, Dewint P, Van den Broeck S, Pauli S, de Schepper H (2019) Rectal cancer surgery: what’s in a name? Acta Gastroenterol Belg 82:67–74 30888757

[14] Birbeck KF, Macklin CP, Tiffin NJ et al (2002) Rates of circumferential resection margin involvement vary between surgeons and predict outcomes in rectal cancer surgery. Ann Surg 235:449–457. 10.1097/00000658-200204000-00001 11923599 10.1097/00000658-200204000-00001 PMC1422458

[15] Votava J, Kachlik D, Hoch J (2020) Total mesorectal excision—40 years of standard of rectal cancer surgery. Acta Chir Belg 120:286–290. 10.1080/00015458.2020.1745529 32200705 10.1080/00015458.2020.1745529

[16] Bregendahl S, Emmertsen KJ, Lous J, Laurberg S (2013) Bowel dysfunction after low anterior resection with and without neoadjuvant therapy for rectal cancer: a population-based cross-sectional study. Colorectal Dis 15:1130–1139. 10.1111/codi.12244 10.1111/codi.12244 23581977

[17] Renehan AG (2016) Techniques and outcome of surgery for locally advanced and local recurrent rectal cancer. Clin Oncol 28:103–115. 10.1016/j.clon.2015.11.006 10.1016/j.clon.2015.11.006 26683258

[18] Pérez Lara FJ, Hebrero Jimenez ML, Moya Donoso FJ, Hernández Gonzalez JM, Pitarch Martinez M, Prieto-Puga Arjona T (2021) Review of incomplete macroscopic resections (R2) in rectal cancer: treatment, prognosis and future perspectives. World J Gastrointest Oncol 13:1062–1072. 10.4251/wjgo.v13.i9.1062 34616512 10.4251/wjgo.v13.i9.1062 PMC8465452

[19] Pai VD, Jatal S, Ostwal V et al (2016) Multivisceral resections for rectal cancers: short-term oncological and clinical outcomes from a tertiary-care center in India. J Gastrointest Oncol 7:345–353. 10.21037/jgo.2016.01.02 27284465 10.21037/jgo.2016.01.02 PMC4880780

[20] Song S, Wu G, Pan H et al (2018) The quality of total mesorectal excision specimen: a review of its macroscopic assessment and prognostic significance. Chronic Dis Transl Med 4:51–58. 10.1016/j.cdtm.2018.02.002 29756123 10.1016/j.cdtm.2018.02.002 PMC5938287

[21] Veltcamp Helbach M, Koedam TWA, Knol JJ et al (2019) Residual mesorectum on postoperative magnetic resonance imaging following transanal total mesorectal excision (TaTME) and laparoscopic total mesorectal excision (LapTME) in rectal cancer. Surg Endosc 33:94–102. 10.1007/s00464-018-6279-9 29967990 10.1007/s00464-018-6279-9 PMC6336750

[22] Nash GM, Weiss A, Dasgupta R, Gonen M, Guillem JG, Wong WD (2010) Close distal margin and rectal cancer recurrence after sphincter-preserving rectal resection. Dis Colon Rectum 53:1365–1373. 10.1007/DCR.0b013e3181f052d4 20847617 10.1007/DCR.0b013e3181f052d4

[23] Cai Y, Li Z, Gu X, Fang Y, Xiang J, Chen Z (2014) Prognostic factors associated with locally recurrent rectal cancer following primary surgery (Review). Oncol Lett 7:10–16. 10.3892/ol.2013.1640 24348812 10.3892/ol.2013.1640 PMC3861572

[24] Ganeshan D, Nougaret S, Korngold E, Rauch GM, Moreno CC (2019) Locally recurrent rectal cancer: what the radiologist should know. Abdom Radiol 44:3709–3725. 10.1007/s00261-019-02003-5 10.1007/s00261-019-02003-5 30953096

[25] Sinaei M, Swallow C, Milot L, Moghaddam PA, Smith A, Atri M (2013) Patterns and signal intensity characteristics of pelvic recurrence of rectal cancer at MRI. Radiographics 33:e171–e187 24025941 10.1148/rg.335115170

[26] Chen F, Ma X, Li S et al (2021) MRI-based radiomics of rectal cancer: assessment of the local recurrence at the site of anastomosis. Acad Radiol 28:S87–S94. 10.1016/j.acra.2020.09.024 33162318 10.1016/j.acra.2020.09.024

[27] Wnorowski AM, Menias CO, Pickhardt PJ, Kim DH, Hara AK, Lubner MG (2019) Mucin-containing rectal carcinomas: overview of unique clinical and imaging features. AJR Am J Roentgenol 213:26–34. 10.2214/AJR.18.20864 30995095 10.2214/AJR.18.20864

[28] Kusters M, Marijnen CAM, van de Velde CJH et al (2010) Patterns of local recurrence in rectal cancer; a study of the Dutch TME trial. Eur J Surg Oncol 36:470–476. 10.1016/j.ejso.2009.11.011 20096534 10.1016/j.ejso.2009.11.011

[29] Waldenstedt S, Bock D, Haglind E, Sjöberg B, Angenete E (2022) Intraoperative adverse events as a risk factor for local recurrence of rectal cancer after resection surgery. Colorectal Dis 24:449–460. 10.1111/codi.16036 34967100 10.1111/codi.16036 PMC9306731

[30] Maas M, Lambregts DMJ, Lahaye MJ et al (2012) T-staging of rectal cancer: accuracy of 3.0 Tesla MRI compared with 1.5 Tesla. Abdom Imaging 37:475–481. 10.1007/s00261-011-9770-5 21674192 10.1007/s00261-011-9770-5 PMC3345180

[31] Jayaprakasam VS, Javed-Tayyab S, Gangai N et al (2021) Does microenema administration improve the quality of DWI sequences in rectal MRI. Abdom Radiol 46:858–866. 10.1007/s00261-020-02718-w 10.1007/s00261-020-02718-w PMC7946648 32926212

[32] Gollub MJ, Arya S, Beets-Tan RG et al (2018) Use of magnetic resonance imaging in rectal cancer patients: Society of Abdominal Radiology (SAR) rectal cancer disease-focused panel (DFP) recommendations 2017. Abdom Radiol 43:2893–2902. 10.1007/s00261-018-1642-9 10.1007/s00261-018-1642-9 29785540

[33] Yoo GS, Park HC, Yu JI (2022) Clinical implication and management of rectal cancer with clinically suspicious lateral pelvic lymph node metastasis: a radiation oncologist’s perspective. Front Oncol 12:960527. 10.3389/fonc.2022.960527 36568216 10.3389/fonc.2022.960527 PMC9768025

[34] Kim YW, Kim NK, Min BS et al (2009) Factors associated with anastomotic recurrence after total mesorectal excision in rectal cancer patients. J Surg Oncol 99:58–64 18937260 10.1002/jso.21166

[35] Müller-Schimpfle M, Brix G, Layer G et al (1993) Recurrent rectal cancer: diagnosis with dynamic MR imaging. Radiology 189:881–889. 10.1148/radiology.189.3.8234720 8234720 10.1148/radiology.189.3.8234720

[36] Gollub MJ, Cao K, Gultekin DH et al (2013) Prognostic aspects of DCE-MRI in recurrent rectal cancer. Eur Radiol 23:3336–3344. 10.1007/s00330-013-2984-x 23979104 10.1007/s00330-013-2984-x

[37] Grosu S, Schafer AO, Baumann T et al (2016) Differentiating locally recurrent rectal cancer from scar tissue: value of diffusion-weighted MRI. Eur J Radiol 85:1265–1270. 10.1016/j.ejrad.2016.04.006 27235873 10.1016/j.ejrad.2016.04.006

[38] Li J, Shiomi A (2021) Lateral lymph node dissection in advanced low rectal cancer treatment. Int J Colorectal Dis 36:2361–2371. 10.1007/s00384-021-03975-x 34152455 10.1007/s00384-021-03975-x

[39] Sluckin TC, Couwenberg AM, Lambregts DMJ et al (2022) Lateral lymph nodes in rectal cancer: do we all think the same? A review of multidisciplinary obstacles and treatment recommendations. Clin Colorectal Cancer 21:80–88. 10.1016/j.clcc.2022.02.002 35339391 10.1016/j.clcc.2022.02.002

[40] Ogura A, Konishi T, Beets GL et al (2019) Lateral nodal features on restaging magnetic resonance imaging associated with lateral local recurrence in low rectal cancer after neoadjuvant chemoradiotherapy or radiotherapy. JAMA Surg 154:e192172. 10.1001/jamasurg.2019.2172 31268504 10.1001/jamasurg.2019.2172 PMC6613303

[41] Fujita S, Mizusawa J, Kanemitsu Y et al (2017) Mesorectal excision with or without lateral lymph node dissection for clinical stage II/III lower rectal cancer (JCOG0212): a multicenter, randomized controlled, noninferiority trial. Ann Surg 266:201–207. 10.1097/SLA.0000000000002212 28288057 10.1097/SLA.0000000000002212

[42] Ogura A, Konishi T, Cunningham C et al (2019) Neoadjuvant (chemo)radiotherapy with total mesorectal excision only is not sufficient to prevent lateral local recurrence in enlarged nodes: results of the multicenter lateral node study of patients with low cT3/4 rectal cancer. J Clin Oncol 37:33–43. 10.1200/JCO.18.00032 30403572 10.1200/JCO.18.00032 PMC6366816

[43] Yokoyama S, Takifuji K, Hotta T et al (2014) Survival benefit of lateral lymph node dissection according to the region of involvement and the number of lateral lymph nodes involved. Surg Today 44:1097–1103. 10.1007/s00595-013-0815-y 24370948 10.1007/s00595-013-0815-y

[44] Kanemitsu Y, Komori K, Shida D et al (2017) Potential impact of lateral lymph node dissection (LLND) for low rectal cancer on prognoses and local control: a comparison of 2 high-volume centers in Japan that employ different policies concerning LLND. Surgery 162:303–314. 10.1016/j.surg.2017.02.005 28366499 10.1016/j.surg.2017.02.005

[45] Komori K, Fujita S, Mizusawa J et al (2019) Predictive factors of pathological lateral pelvic lymph node metastasis in patients without clinical lateral pelvic lymph node metastasis (clinical stage II/III): the analysis of data from the clinical trial (JCOG0212). Eur J Surg Oncol 45:336–340. 10.1016/j.ejso.2018.11.016 30477950 10.1016/j.ejso.2018.11.016

[46] Messiou C, Chalmers AG, Boyle K, Wilson D, Sagar P (2008) Pre-operative MR assessment of recurrent rectal cancer. Br J Radiol 81:468–473. 10.1259/bjr/53300246 18347028 10.1259/bjr/53300246

[47] Torkzad MR, Kamel I, Halappa VG, Beets-Tan RG (2014) Magnetic resonance imaging of rectal and anal cancer. Magn Reson Imaging Clin N Am 22:85–112 24238134 10.1016/j.mric.2013.07.007

[48] Furey E, Jhaveri KS (2014) Magnetic resonance imaging in rectal cancer. Magn Reson Imaging Clin N Am 22:165–190 24792676 10.1016/j.mric.2014.01.004

[49] Soldatos T, Andreisek G, Thawait GK et al (2013) High-resolution 3-T MR neurography of the lumbosacral plexus. Radiographics 33:967–987 23842967 10.1148/rg.334115761

[50] Thawait SK, Chaudhry V, Thawait GK et al (2011) High-resolution MR neurography of diffuse peripheral nerve lesions. AJNR Am J Neuroradiol 32:1365–1372 20966057 10.3174/ajnr.A2257 PMC7964353

[51] Wells BJ, Stotland P, Ko MA et al (2007) Results of an aggressive approach to resection of locally recurrent rectal cancer. Ann Surg Oncol 14:390–395 17063304 10.1245/s10434-006-9119-4

[52] Welten VM, Fields AC, Lu P et al (2019) Omental flaps in patients undergoing abdominoperineal resection for rectal cancer. Int J Colorectal Dis 34:1227–1232. 10.1007/s00384-019-03319-w 31123808 10.1007/s00384-019-03319-w

[53] Katsumata K, Mori Y, Kawakita H, Matsuda D, Enomoto M, Aoki T (2009) A study of the incidence of implantation cyst at anastomotic sites after low anterior resection of the rectum with the double stapling technique. Langenbecks Arch Surg 395:465–469. 10.1007/s00423-009-0542-4 19655162 10.1007/s00423-009-0542-4

[54] Sinaei M, Swallow C, Milot L, Moghaddam PA, Smith A, Atri M (2013) Patterns and signal intensity characteristics of pelvic recurrence of rectal cancer at MR imaging. Radiographics 33:E171–E187. 10.1148/rg.335115170 24025941 10.1148/rg.335115170

[55] Akasu T, Yamaguchi T, Fujimoto Y et al (2006) Abdominal sacral resection for posterior pelvic recurrence of rectal carcinoma: analyses of prognostic factors and recurrence patterns. Ann Surg Oncol 14:74–83. 10.1245/s10434-006-9082-0 17061173 10.1245/s10434-006-9082-0

[56] Meyerhardt JA, Mayer RJ (2005) Systemic therapy for colorectal cancer. N Engl J Med 352:476–487. 10.1056/NEJMr40958 15689586 10.1056/NEJMra040958

[57] Mannaerts GHH, Rutten HJT, Martijn H, Groen GJ, Hanssens PEJ, Wiggers T (2001) Abdominosacral resection for primary irresectable and locally recurrent rectal cancer. Dis Colon Rectum 44:806–814. 10.1007/BF02234699 11391140 10.1007/BF02234699

[58] Hagemans JAW, Van Rees JM, Alberda WJ et al (2020) Locally recurrent rectal cancer; long-term outcome of curative surgical and non-surgical treatment of 447 consecutive patients in a tertiary referral centre. Eur J Surg Oncol 46:448–454. 10.1016/j.ejso.2019.10.037 31761506 10.1016/j.ejso.2019.10.037

[59] Moore HG, Shoup M, Riedel E et al (2004) Colorectal cancer pelvic recurrences: determinants of resectability. Dis Colon Rectum 47:1599–1606 15540287 10.1007/s10350-004-0677-x

[60] Sagebiel TL, Viswanathan C, Patnana M, Devine CE, Frumovitz M, Bhosale PR (2015) Overview of the role of imaging in pelvic exenteration. Radiographics 35:1286–1294. 10.1148/rg.2015140127 26172363 10.1148/rg.2015140127

